# Estimation of the cost-effectiveness of apixaban versus vitamin K antagonists in the management of atrial fibrillation in Argentina

**DOI:** 10.1186/s13561-015-0052-8

**Published:** 2015-06-26

**Authors:** Mariano Anibal Giorgi, Christian Caroli, Norberto Damian Giglio, Paula Micone, Eleonora Aiello, Cristina Vulcano, Julia Blanco, Bonnie Donato, Joaquin Mould Quevedo

**Affiliations:** 1 Department of Pharmacology, Instituto Universitario CEMIC School of Medicine, Buenos Aires, Argentina; 2 Department of Cardiology. FLENI, Buenos Aires, Argentina; 3 Department of Epidemiology, Hospital “Ricardo Gutierrez”, Buenos Aires, Argentina; 4 Department of Pharmacology, Universidad Austral School of Medicine, Pilar, Argentina; 5 Bristol-Myers Squibb Argentina, Buenos Aires, Argentina; 6 Bristol-Myers Squibb US, Wallingford, CT USA; 7 Pfizer US, New York, NY USA

**Keywords:** Apixaban, Warfarin, Novel oral anticoagulants, Cost-effectiveness

## Abstract

Apixaban, a novel oral anticoagulant which has been approved for the prevention of stroke and systemic embolism in non-valvular atrial fibrillation, reduces both ischemic and haemorrhagic stroke and produces fewer bleedings than vitamin K antagonist warfarin. These clinical results lead to a decrease in health care resource utilization and, therefore, have a positive impact on health economics of atrial fibrillation. The cost-effectiveness of apixaban has been assessed in a variety of clinical settings and countries. However, data from emergent markets, as is the case of Argentina, are still scarce.

We performed a cost-effectiveness analysis of apixaban versus warfarin in non-valvular atrial fibrillation (NVAF) in patients suitable for oral anticoagulation in Argentina. A Markov-based model including both costs and effects were used to simulate a cohort of patients with NVAF. Local epidemiological, resource utilization and cost data were used and all inputs were validated by a Delphi Panel of local experts. We adopted the payer’s perspective with costs expressed in 2012 US Dollars.

The study revealed that apixaban is cost-effective compared with warfarin using a willingness to pay threshold ranging from 1 to 3 per capita Gross Domestic Product (11558 – 34664 USD) with an incremental cost-effectiveness ratio of 786.08 USD per QALY gained. The benefit is primarily a result of the reduction in stroke and bleeding events.

The study demonstrates that apixaban is a cost-effective alternative to warfarin in Argentina.

## Background

Atrial Fibrillation (AF) is one of the most frequent arrhythmias in adult population. It’s estimated prevalence is 1 – 2 % in the general population and increases to 10 % in subjects > 65 years old [1, 2]. It is associated with a 5-fold increase in the risk of stroke and systemic embolic events (i.e. pulmonary embolism and myocardial infarction) [3]. The chance of having a stroke depends on several risk factors which are considered in scores like the CHADS2 (**C**ardiac heart failure, **H**ypertension, **A**ge, **D**iabetes, and **S**troke) [4] or, more recently, the CHA[2]DS[2]-VASC (cardiac failure or ejection fraction <40 %, high blood pressure, age 64 to 74 or ≥75 years, diabetes, previous stroke or transient ischemic attack or thromboembolic events, vascular disease, and female sex) [5–7]. These scores constitute the basis for the decision to use medication in order to reduce embolic risk. Until recently therapeutic options to reduce the risk of stroke in AF included oral vitamin K antagonists (VKAs), warfarin and acenocoumarol, and, in patients who were unsuitable for these drugs, aspirin, alone or in association with clopidogrel. Despite the proven efficacy of VKAs, they have several limitations. The limitations include failure to maintain the treatment range (an International Normalized Ratio between 2.00 to 3.00), which results in needing to perform regular coagulation tests and many drug-drug interactions which are cause of the underutilization of VKAs [8, 9]. A measure of the quality of anticoagulation is the time in treatment range (TTR) that indicates the time spent between an INR 2.00 to 3.00. The limitations resulted in the goal to develop alternative treatment options. New Oral Anticoagulants (NOACs), which have unique pharmacodynamic and pharmacokinetic features that result in more stable and predictable anticoagulant effect [10] are recent treatment options to reduce the risk of stroke in AF**.** Currently, there are four NOACs (dabigatran, apixaban, rivaroxaban, and edoxaban) that completed phase III research programs and proved their safety and efficacy [11]. All of these assets except edoxaban have received medical approval for the use in AF in both the United States and Europe. Apixaban, an oral factor Xa inhibitor is the most recent compound to receive medical approval for the prevention of thrombotic events in AF in US and Europe. In one clinical trial for apixaban, ARISTOTLE, Apixaban demonstrated that it is superior to dose-adjusted warfarin in patients suitable for oral anticoagulants [12]. ARISTOTLE revealed a 21 % relative risk reduction in the primary efficacy endpoint (stroke or systemic embolism) and a 31 % relative risk reduction in the safety endpoint (major bleeding). Apixaban was also compared with aspirin in patients who are unsuitable for oral anticoagulation in the AVERROES trial [13]. In this study, apixaban demonstrated a 55 % relative risk reduction in the primary efficacy endpoint (stroke or systemic embolism).

Beyond their efficacy and safety profile, the decision for adopting apixaban by health care decision-makers has been supported by several health economic evaluations. Apixaban received a positive assessment by National Institute of Health Care and Excellence (NICE) in 2013 [14] and several cost-effectiveness analysis have been published revealing that apixaban, compared to either warfarin or aspirin, is a cost-effective alternative given a variety of health care settings [15, 16]. Given that the estimates of about 290,000 patients with AF are suitable for oral anticoagulants in Argentina [17–20], it is important to know the economic impact of new treatment options. The aim of the study is to assess the cost-effectiveness of apixaban versus VKAs in Argentina in order to provide local data for decision-makers.

## Methods

We performed an adaptation of a Markov-based cost-effectiveness model previously submitted by the manufacturers of apixaban (Bristol-Myers Squibb and Pfizer) to NICE in the UK including local epidemiological and clinical data.

### Model

**Fig. 1:**
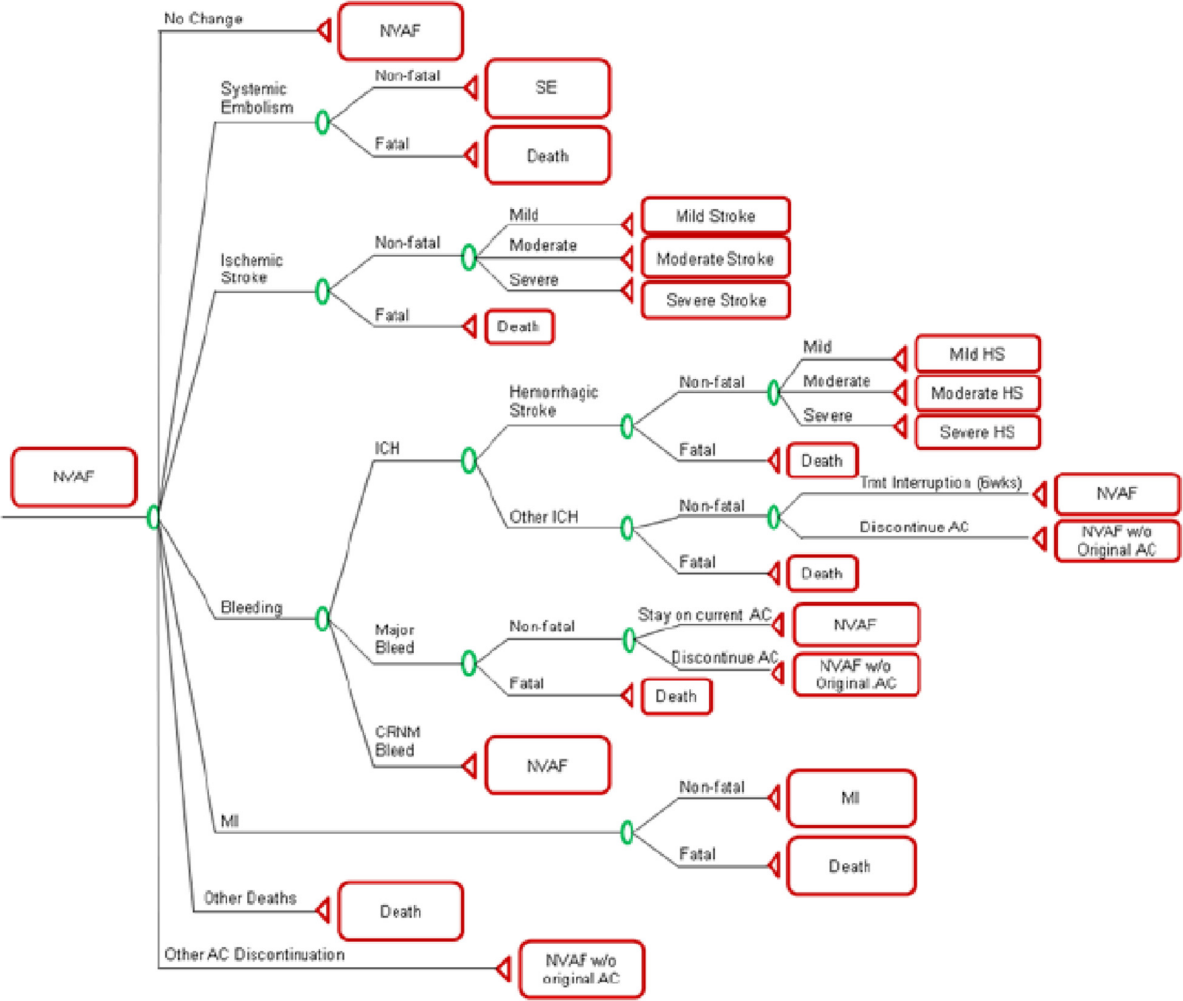
Non-valvular Atrial Fibrillation decision-tree used in the model

The model was developed according to Good Modeling Practices [21], a detailed description is available elsewhere [14, 15]. The model allows a comparison of apixaban against currently available treatment options, including: warfarin, aspirin, dabigatran, rivaroxaban, and aspirin + clopidogrel. For the present analysis we report data for apixaban versus warfarin for patients suitable for oral anticoagulant therapy. The model includes 18 mutually exclusive health states for a hypothetical cohort of patients with non-valvular atrial fibrillation (NVAF) considering the occurrence of stroke (both ischemic or heamorrhagic), systemic embolism (myocardial infarction, pulmonary embolism), bleeding (intracranial, major bleeding, clinically relevant non-major bleeding) and death (Fig. 1). Transition probabilities between health states were derived from the ARISTOTLE trial [12] and from the life expectancy table for Argentina obtained from the World Health Organization. [22] Patients were followed for a lifetime horizon with 6 week cycles, with only allowing for one event per cycle.

### Population

The model considered a hypothetical cohort of 1000 patients with AF suitable for the use of oral anticoagulants. Demographics and baseline stroke risk for the cohort (based on the CHADS2 score) were obtained from published reports (Table 1) [18–20, 23] and expert’s opinions obtained during the Delphi Panel. Anticoagulation quality for VKA users was considered using the average time in therapeutic range (TTR) [24] for centers in Argentina [Table 1] [25, 26].

**Table 1:** Characteristics of the population considered in the model

Population characteristic’s		Source
Gender		[19]
Male	52.4 %	
Female	47.6 %	
Mean Age		[19, 20]
Male	67 years	
Female	73 years	
CHADS2		
0	10.3 %	[18]
1	30.6 %	[18]
2	27.0 %	[23]
3	12.0 %	[23]
≥4	18.1 %	*
Average CHADS2	2.2	
Anticoagulation Control in Centers in		
Argentina (median cTTR)	51 %	[24, 25]
cTTR < 52.38 %		
52.38 % - 66.02 %	22 %	
66.03 % - 76.51 %	22 %	
cTTR ≥ 76.51 %	5 %	

*Assumption based on data from DiTomasso et al. [18]

### Clinical event risks and management

The risk and types of clinical events included in the model are presented in Table 2. Risks considered in the analysis were taken from the ARISTOLE trial [12]. Ischemic stroke risk was adjusted per each decade of life by a factor of 1.40 [27]. Due to inter-countries variation in medical management and treatment patterns, a Panel of Experts was convened using a Delphi method [28]. Two set of experts composed of 6 neurologists and 7 hematologists, representing the three health subsectors from Argentina (public, worker´s unions health care, and private), were consulted about clinical characteristics of patients with AF, treatment patterns, preferences for treatment change in case of bleeding events and resource utilization. All answers were revised in an open discussion and a final set of data was obtained.

**Table 2:** Type and risks of clinical events included in the model *(reported per 100 patient/years)*

Events	Apixaban	Warfarin	Source
Ischemic stroke risk by CHADS2			[15]
Mean	0,962	1,064	
CHADS_2_ score 0	0,521	0,458	
CHADS_2_ score 1	0,521	0,458	
CHADS_2_ score 2	0,950	0,934	
CHADS_2_ score 3	1,534	1,944	
CHADS_2_ score 4	1,534	1,944	
CHADS_2_ score 5	1,534	1,944	
CHADS_2_ score 6	1,534	1,944	
Systemic embolism	0.090	0.100	[12]
Hemorrhagic stroke and Intracranial Hemorrhage	0.330	0.800	[12]
Other major bleeding	1.790	2.270	[12]
Clinically Relevant Non-Major Bleeding	2.083	2.995	[15]
Myocardial Infarction	0,530	0,610	[12]
Other hospitalizations due to cardiovascular disease	10.460	10.460	[15]
Recurrent ischemic stroke	4.103	4.103	[43]
Recurrent hemorrhagic stroke	3.00	3.00	[43]

### Utilities

Currently there is no local data regarding quality of life associated with AF, stroke or any other clinical outcome of interest. We therefore used the values from a UK catalogue of EQ-5D score [29] for each health state (Table 3).

**Table 3:** Utility and utility decrements associated with health states and treatments included in the model (measured by EQ-5D) from reference 30

Health State	Utility (SE)
Non-valvular atrial fibrillation	0.7270 (0.0095)
Stroke (ischemic or hemorrhagic)	0.6151 (0.0299)
*Mild*	0.5646 (0.0299)
	0.5142 (0.0299)
*Moderate*	
*Severe*	0.6151 (0.0299)
Myocardial infarction	0.5646 (0.0299)
	0.6265 (0.0299)
*Females*	
*Males*	
Systemic embolism	
Transient health states/anticoagulation use	Utility decrements (SE/95 % CI)
Other intracranial haemorrhage	0.1511 (0.0401)
Other major	0.1511 (0.0401)
Clinically relevant non-major bleed	0.0582 (0.0173)
Other cardiovascular hospitalization	0.1276 (0.0259)
Use of Apixaban or aspirin	0.0020 (0.00-0.04)
Use of Warfarin	0.0120 (0.00-0.08)

### Costs and resource utilization

Direct costs and resource utilization for each health state included in the model were obtained from local data sources (Table 4) [30–33]. For data that was not available through a published report, we obtained the data from a Health Resource Cost Database. For the economic evaluation, we considered the perspective of the payer. Because the Argentinean health system comprises three health subsectors (public, worker’s unions health care, and private), each one with its own resource utilization pattern and prices, we reported cost as a weighed mean. For each item we considered the price for each health subsector multiplied by the proportion of subjects covered by the sector: 52.5 % by the public sector, 38.8 % by the worker’s union sector and 9 % by the private sector [34]**.** For example, the reported cost of stroke is: *cost for the public sector x 0.522 + cost for the worker’s union sector x 0.388 + price for the private sector x 0.09*.

**Table 4:** Drug and Event costs (in 2012 US Dollars)

Item	Cost (USD) [min-max]	Unit	Duration of the event	Source
Drugs				
*Apixaban 5 mg (BID)*	1.49	Per day	--	[30]
*Warfarin (5 mg/day average dose)*	0.15	Per day		
Monitoring Visit *(applicable to warfarin only)*	11.85 [9.24-14.45]	Per visit	--	*
Routine Care	1.11 [0.86-1.35]	Per visit	--	*
Stroke *(excluding hemorrhagic stroke)*				
*Mild*				[31, 32]
*Acute Care*	1450.33 [1131.25-1769.4]	Per episode	2 weeks	
*Long-term Maintenance*	1110.20 [865.95-1354.44]	Per month	Lifetime	
*Moderate*				
*Acute Care*	2813.25 [2194.33-3432.16]	Per episode	2 weeks	[31, 32]
		Per month	Lifetime	
*Long-term Maintenance*	1110.20 [865.95-1354.44]			
*Severe*				
*Acute Care*	4084.26 [3185.72-4982.79]	Per episode	2 weeks	[31, 32]
		Per month	Lifetime	
*Long-term Maintenance*	1110.20 [865.95-1354.44]			
*Fatal Ischemic Stroke*	2813.25 [2194.33-3432.16]	Per episode		
Heamorrhagic Stroke				
*Mild*				
*Acute Care*	3740.68 [2917.73-4563.62]	Per episode	2 weeks	[31, 32]
*Long-term Maintenance*	1110.20 [865.95-1354.44]	Per month	Lifetime	
*Moderate*				[31, 32]
*Acute Care*	6731.00 [5250.18 – 8211.82]	Per episode	2 weeks	
*Long-term Maintenance*	1110.20 [865.95-1354.44]	Per month	Lifetime	
*Severe*				
*Acute Care*	13777.79 [10746.67-16808.9]	Per episode	2 weeks	[31]
*Long-term Maintenance*	1110.20 [865.95-1354.44]	Per month	Lifetime	
*Fatal Heamorrhagic Stroke*	6731.00 [5250.18-8211.82]	Per episode		**
Systemic Embolism				
*Acute Care*	2900.04 [2262.03-3538.04]	Per episode	2 weeks	[33]
*Long-term Maintenance*	229.11 [178.70-279.52]	Per month	Lifetime	
Other ICH *(excluding heamorrhagic stroke)*	6622.09 [5165.23-8078.94]	Per episode	--	[31, 33] *
Other Major Bleeds			--	
*(excluding ICH)*				
*GI Bleeds*	3829.17 [2986.75-4671.58]	Per episode		
*Non ICH and Non GI Related Major Bleeds*	3829.17 [2986.75-4671.58]	Per episode		
			--	[33]
			--	[33]
CRNM Bleeds	2055.04 [750.7-1284.28]	Per episode	--	[33]
Myocardial Infarction				
*Acute Care*	2211.52 [1748.00-2797.90]	Per episode	--	[32]
*Long-term Maintenance*	1110.20 [865.95-1354.44]	Per month	Lifetime	[32] *
Other CV Hospitalization	2211.52 [1139.70-1797.70]	Per episode	--	[32]

*GI bleeds* gastrointestinal bleeds; *ICH* intracranial hemorrhage; *CRNM bleed* clinically relevant non-major bleeds

*Based on a local Health Resource Cost Data Base

**we assumed that fatal stroke (both ischemic or haemorrhagic) has a cost equivalent to a moderate stroke(both ischemic or haemorrhagic) reported by Christensen et al. [31]

Drug costs were obtained from local formularies and adapted for each health subsector [30]. All prices were updated to the last quarter of 2012 and expressed in 2012 US Dollars (USD).

### Economic analyses

We estimate the clinical effectiveness of apixaban versus warfarin in terms of events per 1000 treated patients using a lifetime scenario. Results were expressed as the incremental cost-effectiveness ratio (ICER) considering a cost effectiveness threshold between 1 to 3 per capita GDP (Gross Domestic Product) accordingly with the WHO-CHOICE recommendation [35]. The 2011 per capita GDP for Argentina was estimated as 11,558 USD [36], therefore the willingness to pay for every incremental QALY ranges from 11,558 USD to 34,674 USD. A 5 % discount rate was applied to both costs and events, as recommended by regional regulations in 2009 [37].

Deterministic sensitivity analysis was performed and presented as tornado graphic in order to assess the influence of key variables over the incremental cost-effectiveness ratio.

Probabilistic sensitivity analysis was conducted changing model’s parameters. Two thousand simulations were ran and plotted in a cost-effectiveness plane considering cost and QALY.

Sensitivity analyses based on the use of other AVK agent (acenocoumarol), cost’s discount rate and time horizon were also performed.

## Results

### Clinical effectiveness

In a VKA suitable cohort of 1000 patients with NVAF, compared with warfarin, the use of apixaban resulted in 24 fewer strokes (including first and recurrent ischemic and hemorrhagic) or systemic embolism, 41 fewer major bleeding events (including first and recurrent hemorrhagic stroke, other intracranial hemorrhage and other major bleeds), and 26 fewer cardiovascular-related deaths (Table 5). It is therefore estimated that 56 patients (number needed to treat) should be treated with apixaban over a lifetime in order to avoid one stroke (ischemic and hemorrhagic) compared with warfarin. In addition, apixaban is a better option than warfarin avoiding bleeding events (number needed to harm = 24). The use of apixaban in this target population resulted in an increase of 0.164 life years and 0.172 QALYs (Table 5).

**Table 5:** Clinical events in the cohort of NVAF patients treated with Apixaban and warfarin

		VKA Suitable patients
Number of events (Total population)ischemic strock	Apixaban	Warfarin
*Non-fatal Mild*	80	80
*Non-fatal Moderate*	68	73
*Non-fatal Severe*	27	28
*Fatal*	25	25
TOTAL	200	206
Recurrent Islamic Stroke		
*Non-fatal Mild*	4	4
*Non-fatal Moderate*	6	7
*Non-fatal Severe*	5	6
*Fatal*	4	4
TOTAL	20	21
Hemorrhagic Stroke		
*Non-fatal Mild*	4	7
*Non-fatal Moderate*	6	6
*Non-fatal Severe*	4	6
*Fatal*	9	21
*TOTAL*	23	40
Recurrent Hemorrhagic Stroke		
*Non-fatal Mild*	0	0
*Non-fatal Moderate*	0	0
*Non-fatal Severe*	0	1
*Fatal*	0	0
*TOTAL*	1	2
Systematic Embolism		
*Non fatal*	19	19
*Fatal*	2	2
*TOTAL*	22	21
Other ICH		
*Non fatal*	9	21
*Fatal*	1	3
*TOTAL*	11	24
Other Major Bleeds		
*Non-fatal GI Bleeds*	54	55
*Non-fatal Non ICH or Non GI Related Major Bleeds Fatal*	88	98
*Fatal*	3	3
*TOTAL*	145	155
Clinically Relevant Non-Major Bleeds	252	287
*MI*		
*Non fatal*	67	67
*Fatal*	8	8
*TOTAL*	75	75
*Other CV Hospitalization*	1.060	1.020
Other Treatment Discontinuation	579	591
*Deaths*		
*Event Related (acute)*	51	65
*Event related (death due to stroke, HS,MI, SE)*	273	285
*Other*	676	650
*TOTAL*	1.000	1.000

### Costs

Table 6 presents the incremental costs of the use of apixaban compared with warfarin using a lifetime horizon. In every situation, the result falls below the willingness to pay threshold of 11558 USD (equivalent to one per capita GDP).

**Table 6:** Summary results of the cost-effectiveness analysis

	VKA Suitable Patients
	*Apixaban-Warfarin*
Net Cost	USD 135,06
Net Life Years	0,164
Net QALYs	0,172
ICER	
Cost per Life Year gained	USD 823,29
Cost per QALY gained	USD 786,08
Cost per Stroke Avoided (Ischemic and Hemorrhagic)	USD 5.422,01
Cost per Bleed Avoided (ICH including HS and Major Bleed)	USD 3.268,66

### Sensitivity analysis

**Fig. 2:**
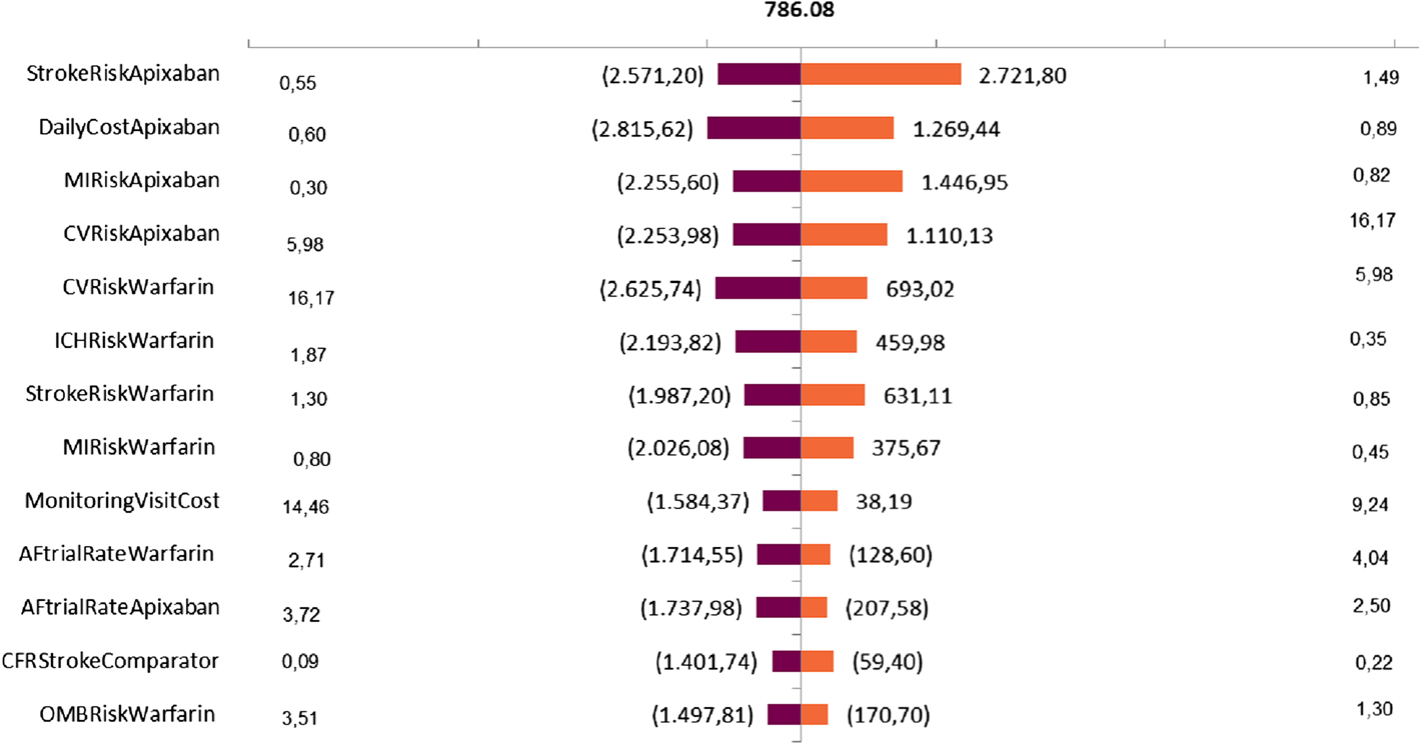
Tornado sensitivity analysis of the incremental cost-effectiveness ratio of Apixaban compared with warfarin. The solid vertical line represents the base-case incremental cost-effectiveness ratio. In the vertical lines are depicted the range obtained for a variable while the others are constant. MI: myocardial infarction; CV: cardiovascular hospitalization; ICH: intracranial hemorrhage; Monitoring visit: applied only to patients treated with warfarin; AFtrialRate: treatment´s discontinuation rate; OMB: other major bleed

**Fig. 3:**
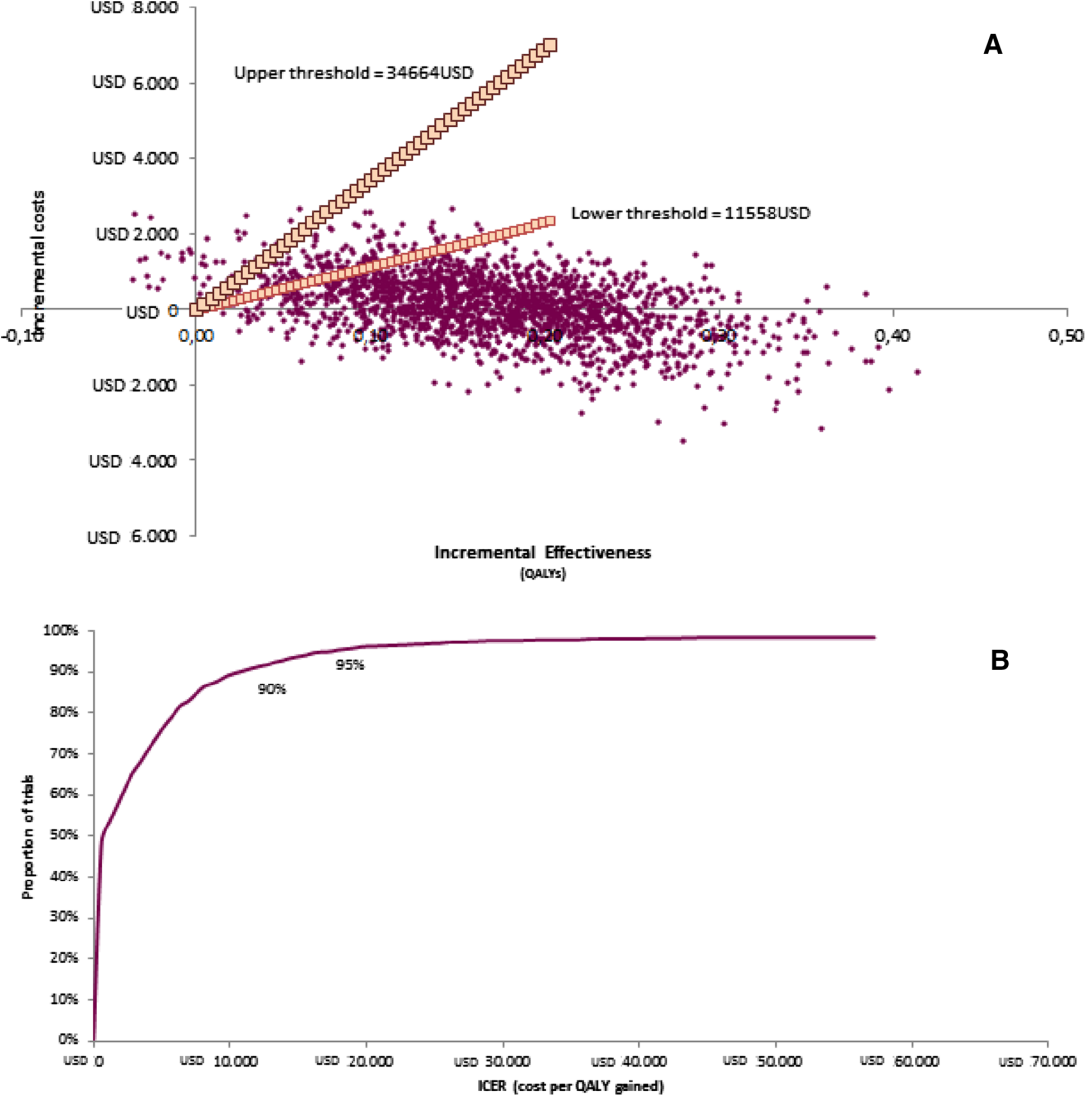
**a** Probabilistic sensitivity analysis of the incremental cost-effectiveness ratio of Apixaban compared with warfarin. **b** Acceptability curve for Apixaban compared with warfarin. **a** Upper threshold per QALY gained: 34664 USD; Lower Threshold per QALY gained: 11558 USD. **b** Probability of being accepted as a cost-effectiveness alternative considering the upper and lower thresholds showed in figure 3a

A one way sensitivity analysis (Fig. 2) revealed that the risk of stroke with apixaban is the main contributor to the cost-effectiveness. In Fig 3a and b are depicted the results of the probabilistic sensitivity analysis. The analysis presents that apixaban is more effective and more costly than warfarin. Considering a willingness to pay threshold of 11,558 USD per QALY gained, apixaban is a cost-effective alternative in 90 % of the cases. Using the upper threshold of 34,664 USD apixaban has a 95 % probability of being a cost-effective alternative compared to warfarin.

Because warfarin is not the only AVK available in Argentina, we perform a separate analysis considering the cost for acenocoumarol. In this case the cost per life year gained was 745.20 USD and the cost per QALY gained was 711.52 USD.

We also assessed if changes in costs’ discounting rate would alter our results. Because there is no specific long-term investment rate in Argentina we run the analysis raising the discount rate to 10 % and 15 %. For the 10 % cost discount rate the cost per life year gained was 798.26 USD and the cost per QALY gained was 762.18 USD. For the 15 % cost discount rate the cost per life year gained was 811.28 USD and the cost per QALY gained was 774.61 USD.

Finally, we assessed different scenarios based on time horizon. For a 2 year scenario the cost per life year gained was 17043.00 USD and the cost per QALY gained was 6153.35 USD. For a 5 year scenario cost per life year gained was 3624.11 USD and the cost per QALY gained was 2234.97 USD.

## Discussion

Our study estimated that apixaban is a cost-effective option, compared to warfarin, for the management of NVAF with an ICER of 786.08 USD/QALY. Local epidemiological data regarding AF in Argentina revealed that subjects suitable for oral anticoagulants are older and have a higher stroke risk (assessed by the CHADS2 score) than patients included in clinical trials [12]. We also found that the TTR is less than optimal in more than two third of the centers in Argentina. These findings are particularly relevant when the clinical effectiveness of apixaban is assessed versus vitamin K antagonist warfarin. In fact, considering the results of the model, apixaban use resulted in fewer thrombotic and bleeding events than warfarin, leading to less health resource utilization. Therefore, in spite of the much higher drug cost for apixaban, the strategy of adopting this novel anticoagulant results in an incremental cost of only 135 USD in a lifetime scenario. Even considering the cost for acenocoumarol instead of warfarin in a sensitivity analysis, the ICERs obtained were consistent with the cost-effectiveness of apixaban over all other options.

The definition of a willingness to pay threshold for incremental effectiveness is a matter of debate in countries that lack a defined value, as happens in the United Kingdom [38]. The adoption of the WHO Choice rule, using the GDP per capita as a parameter to set thresholds, is a valuable strategy in developing countries [35]. In the case of Argentina, which has three different payers’ sub-sectors, this method for establishing a threshold provides a wide reference for decision-makers. Therefore, the use of sensitivity analysis as a way to manage uncertainty provides a range of costs-effectiveness ratios that improves availability of data for decision-makers [39].

Our results are concordant with published cost-effectiveness analysis from other countries reporting that apixaban is a cost-effectiveness alternative. Canestaro et al. [16] reported for the United States, that apixaban is the optimal anticoagulant resulting in a net effectiveness increment of 0.41 QALY compared with warfarin. Accordingly, using the same model, Dorian et al. revealed that in the United Kingdom, apixaban improved both life expectancy and quality adjusted-life years compared to warfarin. Both studies, which used different cost data, revealed that apixaban is the most cost-effective therapeutic alternative.

The development of local data is of capital importance in the process of health technology assessment. Moreover, considering that most physicians in Argentina require that a medical technology is fully tested before adopting it [40], the availability of data which includes both clinical and economic aspects will certainly contribute to the decision making process.

Our study has many limitations. Local data are scarce and it is difficult to obtain needed data in published reports. This reflects problems regarding scientific publications in Argentina [41]. As a consequence, the relative weight of local experts’ opinion in validating information is much higher than in other countries. This issue has many potential consequences over results. The introduction of biases in expert’s responses (such as anchoring effects, absolute and relative judgements) are one of the most important factors to be taken into account when results are considered [42]. Finally, in recent years Argentina developed economic instability which could represent an objection with the discount rate adopted in this report despite that there have not been any discounting modifications in regional recommendations for conducting health economic evaluations.

## Conclusions

In our study, using local epidemiological estimates and based on randomised clinical trials data, apixaban resulted a cost-effectiveness alternative to warfarin according to local willingness to pay thresholds.
